# The Variation Characteristic of Sulfides and VOSc in a Source Water Reservoir and Its Control Using a Water-Lifting Aerator

**DOI:** 10.3390/ijerph13040427

**Published:** 2016-04-15

**Authors:** Jian-Chao Shi, Ting-Lin Huang, Gang Wen, Fei Liu, Xiao-Peng Qiu, Bao-Shan Wang

**Affiliations:** 1 Key Laboratory of Northwest Water Resource, Environment and Ecology, MOE, Xi’an University of Architecture and Technology, Xi’an 710055, China; shi_jch@163.com (J.-C.S.); liufei_tsusc@yeah.net (F.L.); qxp2015@163.com (X.-P.Q.); wbs@mail.lzjtu.cn (B.-S.W.); 2 Shaanxi Key Laboratory of Environmental Engineering, Xi’an University of Architecture and Technology, Xi’an 710055, China

**Keywords:** source water, water quality improvement, sulfides, volatile organic sulfur compounds, water-lifting aerator, reservoir, dissolved oxygen

## Abstract

Sulfides and volatile organic sulfur compounds (VOSc) in water are not only malodorous but also toxic to humans and aquatic organisms. They cause serious deterioration in the ecological environment and pollute drinking water sources. In the present study, a source water reservoir—Zhoucun Reservoir in East China—was selected as the study site. Through a combination of field monitoring and *in situ* release experiments of sulfides, the characteristics of seasonal variation and distribution of sulfides and VOSc in the reservoir were studied, and the cause of the sulfide pollution was explained. The results show that sulfide pollution was quite severe in August and September 2014 in the Zhoucun Reservoir, with up to 1.59 mg·L^−1^ of sulfides in the lower layer water. The main source of sulfides is endogenous pollution. VOSc concentration correlates very well with that of sulfides during the summer, with a peak VOSc concentration of 44.37 μg·L^−1^. An installed water-lifting aeration system was shown to directly oxygenate the lower layer water, as well as mix water from the lower and the upper layers. Finally, the principle and results of controlling sulfides and VOSc in reservoirs using water-lifting aerators are clarified. Information about sulfides and VOSc fluctuation and control gained in this study may be applicable to similar reservoirs, and useful in practical water quality improvement and pollution prevention.

## 1. Introduction

Reservoirs have gained increasing importance as sources of drinking water for urban areas. In reservoirs, a three-layer temperature distribution tends to form in the summer due to the long hydraulic retention time and poor water convection [1]. Such thermal stratification of reservoir water blocks the re-aeration pathway of the bottom-most water. Chemical and biological processes may consume dissolved oxygen in the water, leading to the formation of anaerobic environment in the hypolimnion [2,3]. As anaerobic conditions at the profundal sediment-water interface progresses, the bottom-most water accumulates a range of compounds that exacerbate eutrophication, are toxic to aquatic biota, and limit the potential for potable water use [4].

Many researchers have studied the formation of thermal stratification and problems caused by hypolimnetic hypoxia [5,6,7,8,9]. Their studies mainly concentrated on the prediction of water temperature distribution, spatiotemporal evolution of nutrients and their ecological impacts, effects on phytoplankton blooms, *etc.* [10,11,12]. However, there is little research on sulfur cycling in reservoirs where the concentration of sulfides is low. The cycling of sulfur is dynamic, both spatially and temporally, and it strongly influences many biogeochemical reactions in sediments [13]. The presence of large amounts of organic matter in the sediments typically results in oxygen depletion, followed by the production of sulfides by sulfate-reducing bacteria (SRB) [14]. The sulfides produced by SRB may, then, chemically react with metals to form insoluble sulfides, such as FeS and FeS_2_ [15,16]. In fresh water systems, including deep water reservoirs, sulfur pollution comes from the following compounds: sulfides including the soluble H_2_S, HS^−^ and S^2−^, acid-soluble metal sulfides, ionized organic and inorganic sulfur compounds in the suspension [17], and volatile organic sulfur compounds (VOSc) which include dimethyl sulfide, carbonyl sulfide, and carbon disulfide, *etc.* [18]. The sulfides in water are toxic to aquatic organisms, as they can react with cytochrome, oxidase, and disulfide bonds (-S-S-) to affect cell oxidation process and cause cell hypoxia. Hydrogen sulfide (H_2_S) and its oxidation product, sulfuric acid, may corrode water pipelines. In addition, hydrogen sulfide produces the characteristic rotten egg smell. The soluble sulfides may combine with organic matter in the water to form VOSc, which have bad odors [19]. Sulfides and VOSc seriously affect the aquatic ecology and the function of the reservoir as a drinking water source. The sulfide limit specified by the national quality standard for raw water (Level 3) is 0.20 mg·L^−1^ [20], and threshold value of VOSc is gammagrade [21]. Hence, it is very important to understand their spatial and temporal variations in reservoir water. 

Water-lifting aeration technology has been developed for *in situ* water quality improvement and eutrophication control of lakes and reservoirs. The water-lifting aerator (WLA) combines the advantages of the air-lifting aerator and hypolimnion aeration, which can mix the upper and lower layers of water and directly oxygenate water [22,23,24]. Over recent years, our research group has carried out systematic and comprehensive theoretical studies on the structure and performance of WLAs, as well as applied studies of their control of eutrophication and inhibition of algal growth and nutrient-release from sediments in two reservoirs in North China (Shanxi Fenhe Reservoir and Shaanxi Jinpen Reservoir). However, the effect and principle of controlling sulfides and VOSc in reservoirs using WLA remain poorly understood.

A major motivation for this study is the deterioration of water quality in summer because of thermal stratification, which has been observed in many reservoirs and lakes around the world [25,26,27]. The Zhoucun Reservoir in East China was selected as the study site. It is an important source water reservoir of Zaozhuang, a medium-sized city nearby. Three interrelated subjects are discussed in this article: (1) field monitoring data are used to analyze the migration and distribution of sulfides and VOSc during thermal stratification in the reservoir; (2) a novel *in situ* release instrument is used to reproduce the sulfides releasing process and find the sources of sulfides and VOSc; and (3) the effect and principle of sulfides and VOSc control in reservoirs using WLAs are presented.

## 2. Materials and Methods

### 2.1. Study Site

**Figure 1:**
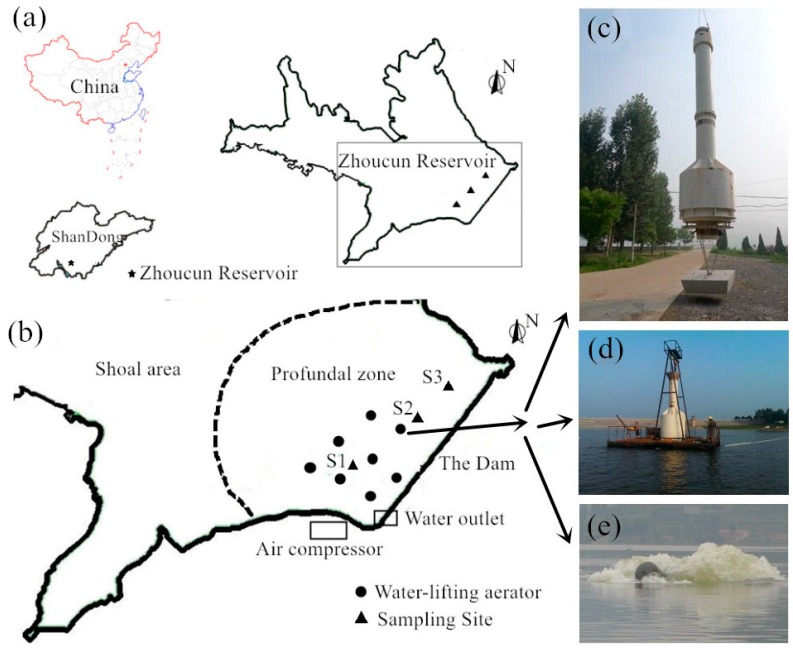
Bathymetric map of the Zhoucun Reservoir with the location of the sampling site (**a**); the layout of the WLAs in the reservoir (**b**) and WLA photos during construction and operation (**c**–**e**) ((**c**) appearance of WLA; (**d**) work platform on the water; and (**e**) surge from the WLA).

**Figure 2:**
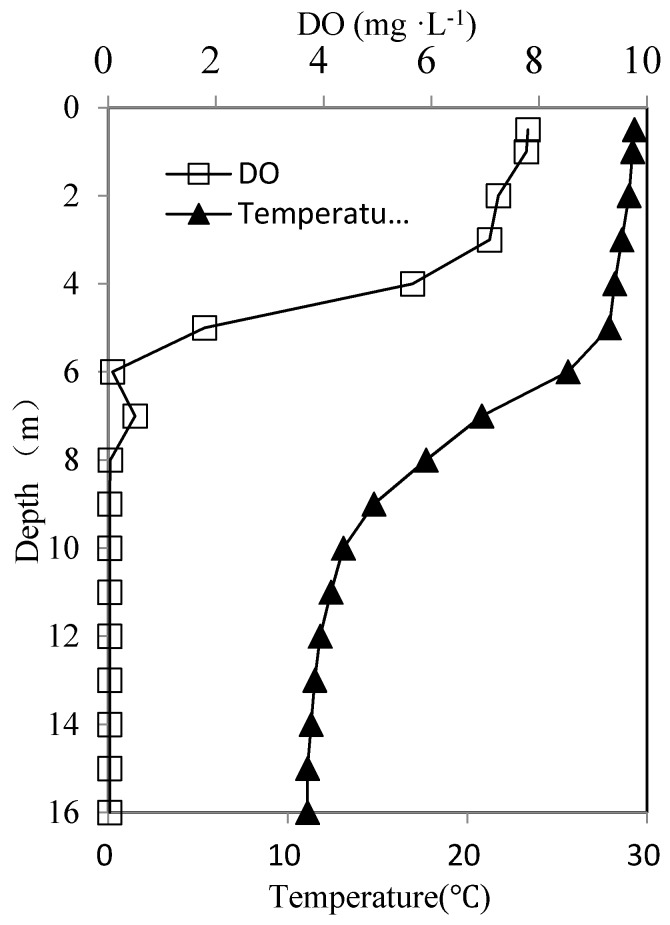
Dissolved oxygen (DO) and temperature profile in the Zhoucun Reservoir at the time of stratification.

The Zhoucun Reservoir (Figure 1a) (34°56′28″–34°58′7″N, 117°39′48″–117°41′21″E) is a eutrophic reservoir, and an important source of water for the nearby city. It is located on the south border of Yimeng Mountain in East China. It has a mean depth of 16 m and a drainage area of 121 km^2^. In its drainage area, there are only a few small poultry farms; no coking, paper milling, leather processing, or other industrial wastewater is discharged into the reservoir. The reservoir has been used for cage fish farming for more than 30 years, which results in its eutrophication in recent years [28]. From April to November 2014, hypoxia due to stratification was one of the environmental problems in the bottom water (from 6 m to the sediments surface) of the Zhoucun Reservoir (Figure 2). 

### 2.2. Water Sampling and Analysis

In this study, three sampling sites along the dam (shown in Figure 1b) were picked in the deepest area of the main reservoir. Water samples were obtained with a 2.0 L sterile Van-Dorn bottle at depth intervals of 2.5 m (from 0.5 m below the water-air interface to 0.2 m above the sediment). The depth of the water is 16 m, and the middle layer sample was obtained at 7.5 m above the top of the sediment layer.

Water temperature (T), dissolved oxygen (DO), and oxidation reduction potential (ORP) were measured *in situ* with increments of 0.5 m, using a multiparameter water quality analyzer (Hydrolab DS5, Hach, Loveland, CO, USA). For the DO sensor, the detection limit is 0.01 mg·L^−1^ and its sensitivity is ±0.2 mg·L^−1^, and for the ORP sensor, limits of detection is 1 mV and its sensitivity is ±20 mV. The concentration of sulfides was determined using the metheylene blue method [17]. The concentration of VOSc in the water was determined by gas chromatography-mass spectrometry (GC-MS), the limits of detection is 0.6–5 μg·L^−1^ [29,30,31]. VOSc were collected with a Tekmar 3100 purge and trap system from Tekmar (Cincinnati, OH, USA). The purged temperature was 40 °C, using He (40 mL·min^−1^ for 11 min) and a VOCARB 4000 trap column (Supelco, Bellefonte, PA, USA) with cryo focusing at −180 °C. Gas chromatography was performed on a Agilent 7890A coupled to a 5975C mass spectrometer (Agilent, Palo Alto, CA, USA) using the capillary column HP-5MS (30 m × 0.25 mm × 0.25 μm, Palo Alto, CA, USA). The temperature program was adjusted to the separation of the high volatile compounds: 35 °C (15 min), 10 °C·min^−1^ to 200 °C (10 min). The carrier gas is He (1 mL·min^−1^). The mass spectrometer was run in the scan mode for all measurements (Scan mode: 30–300 Dalton, ionization: EI+). Standard samples were purchased from Sigma-Aldrich (Steinheim, Germany).

### 2.3. In Situ Release Experiment

**Figure 3:**
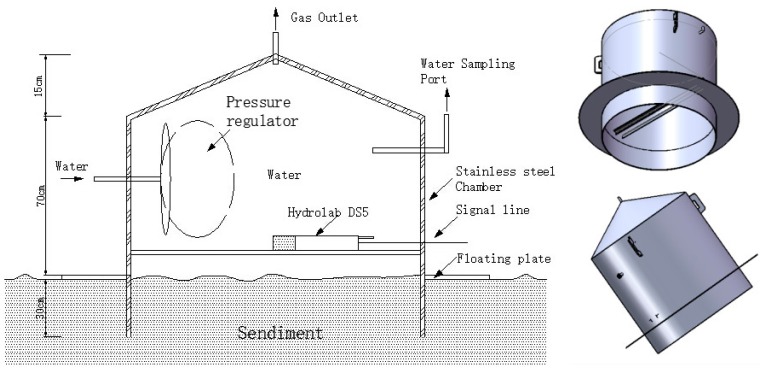
Schematic of the *in situ* experimental chamber.

Experiments have been carried out to verify that the anoxic water was caused by oxygen consumption by the sediments. The sediments’ oxygen consumption was measured on-site in the reservoir. An *in situ* experimental chamber was constructed as shown in Figure 3, and installed at the bottom of the reservoir. The chamber contains a certain volume of bottom water, and maintains the natural conditions of the water-sediment-microbes multi-phase interface. The main structure of the chamber is a cylinder (D = 1 m, H = 1 m, with 0.3 m deep into the sediments) with a conical cap (H = 0.15 m), and its effective volume is 837 L. DO and ORP were measured with a multiparameter water quality analyzer installed in the experimental chamber. A water sample in the chamber was obtained by a diaphragm pump connected to the sampling port, and sulfide concentration was tested daily.

The experiment lasts for 80 days; water temperature in the chamber vary from 6.0–10.5 °C. The sediments’ oxygen consumption rate (SOD) was calculated with the following equation:

(1) $$SOD=\frac{\left(C_{0}-C_{e}\right)\cdot V}{A\cdot t}\cdot \left(\text{mg}\cdot m^{-2}\cdot h^{-1}\right)$$

where C_0_ and C_e_ are the initial and final dissolved oxygen concentrations in mg·L^−1^, V is the water volume in the test chamber in L, A is the cross section area of the test chamber measured in m^2^, and t is the test duration in h [32].

### 2.4. Water Stability Index (RWCS)

In order to determine the stratification of a reservoir, a water stability index (RWCS) was proposed by Padisák in 2003 [33], the equation of RWCS is as follows:

(2) $$RWCS=\frac{D_{b}-D_{s}}{D_{4}-D_{5}}$$

where *D*_s_ is the water density oatf the surface; *D*_b_ is water density at the bottom; *D*_4_ is the water density at 4 °C; *D*_5_ is the water density at 5 °C.

### 2.5. Construction and Operation of Water-Lifting Aeration System

With the support of National Science and Technology Pillar Program, a water-lifting aeration system including eight WLA units was installed in the Zhoucun Reservoir in 2015; the system layout is shown in Figure 1b. The system was set to improve the water quality near the water release tunnel, and prevent anaerobic environment in the lower layer water near the tunnel. More information on the technical parameters of the WLA can be found in the references [34].

The water-lifting aeration system began its operation on 28 August 2015, and stopped on 1 October 2015 when the water was completely mixed. The rate of air supplied to each WLA unit was 1.00–2.00 m^3^·min^−1^. The rate of circulating water flow produced by each WLA was 2.25–4.50 × 10^4^ m^3^·day^−1^ according to the water lifting model in manufacturer specification, and the rate of oxygenation by each aeration chamber was calculated as 35–70 kg·day^−1^ by the oxygenation model, assuming an air supply rate of 1.00–2.00 m^3^·min^−1^ [34]. Photos of the WLAs during construction and operation are shown in Figure 1c–e.

### 2.6. Data Analysis

SPSS 19.0 (IBM SPSS, Chicago, CA, USA) was used to conduct the Pearson correlation analysis. The correlation between VOSc and the concentration of sulfides were tested by liner regression analysis and *t*-tests.

## 3. Results and Discussion

### 3.1. Seasonal and Spatial Variation of Sulfides and VOSc in Reservoir Water

**Figure 4:**
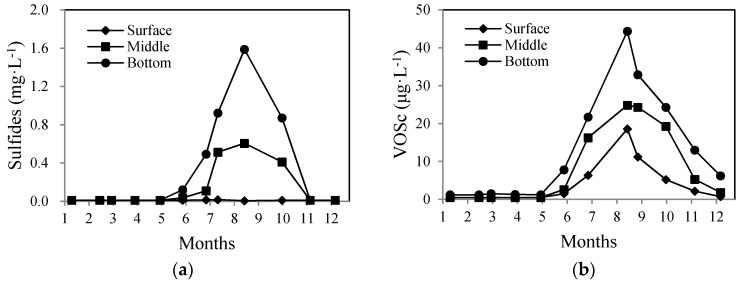
Seasonal variation of sulfides (**a**) and VOSc; (**b**) at different water layer in the Zhoucun Reservoir.

The seasonal variation of sulfides in different water layers in the Zhoucun Reservoir during 2014 is shown in Figure 4a, with the sulfides concentration starting to increase in the middle of June, and reaching the highest concentration of 1.59 mg·L^−1^ in the bottom layer in August. The amount of sulfides then decrease gradually and became undetectable after November. A similar variation pattern exists in the middle water layer, but with a lower peak sulfide concentration (0.61 mg·L^−1^) in August. During June to November 2014, sulfide concentrations in the middle and bottom water layers far exceeded the limit of 0.20 mg·L^−1^ specified by the national quality standard for raw water (Level 3). The sulfides in the surface water layer could not be monitored. This is because the thermocline was at a depth of approximately 6 m (Figure 2). Water in the hypolimnion below 6 m in depth exhibited hypoxia (DO = 0 mg·L^−1^), and sulfides accumulate. The existence of the thermocline block the pathway of sulfides in the hypolimmnion to migrate to the surface water.

In contrast to sulfides, VOSc can be monitored in the surface water, possibly due to their volatile property. Figure 4b shows the variation of VOSc in the surface, middle, and bottom water layers of the reservoir. It can be clearly seen that VOSc concentration started to increase in June, the same time the sulfides concentration started to increase. The worst VOSc pollution appeared in August, with peak values of 18.59 μg·L^−1^, 24.84 μg·L^−1^, and 44.37 μg·L^−1^ in the surface, middle, and bottom layers, respectively. Afterwards, VOSc concentration decreased gradually, down to the same level in November as at the start time (June). From December to May the following year, VOSc concentration in the water stayed at low levels, even undetectable in some months. The VOSc concentrations among the three layers of water are in the following order: bottom > middle > surface.

**Figure 5:**
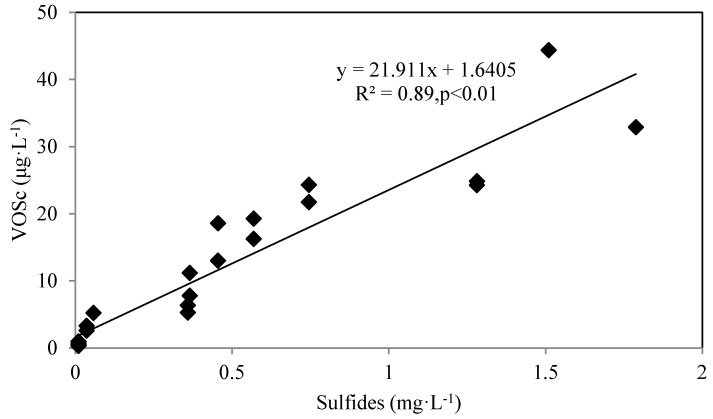
Correlation between VOSc and concentration of sulfides at the bottom of the reservoir.

The production of VOSc in water is affected by many factors, an important one being the concentration of sulfides in the water and sediments [35]. Analyzing the relationship between VOSc and sulfides concentrations (Figure 5), we found they are correlated with each other during summer time (*R*^2^ = 0.89, *p* < 0.01). This suggests the high concentration of sulfides is partially responsible for the increase of VOSc in the water.

### 3.2. Effect of Thermal Stratification on the Concentration of Sulfides

**Figure 6:**
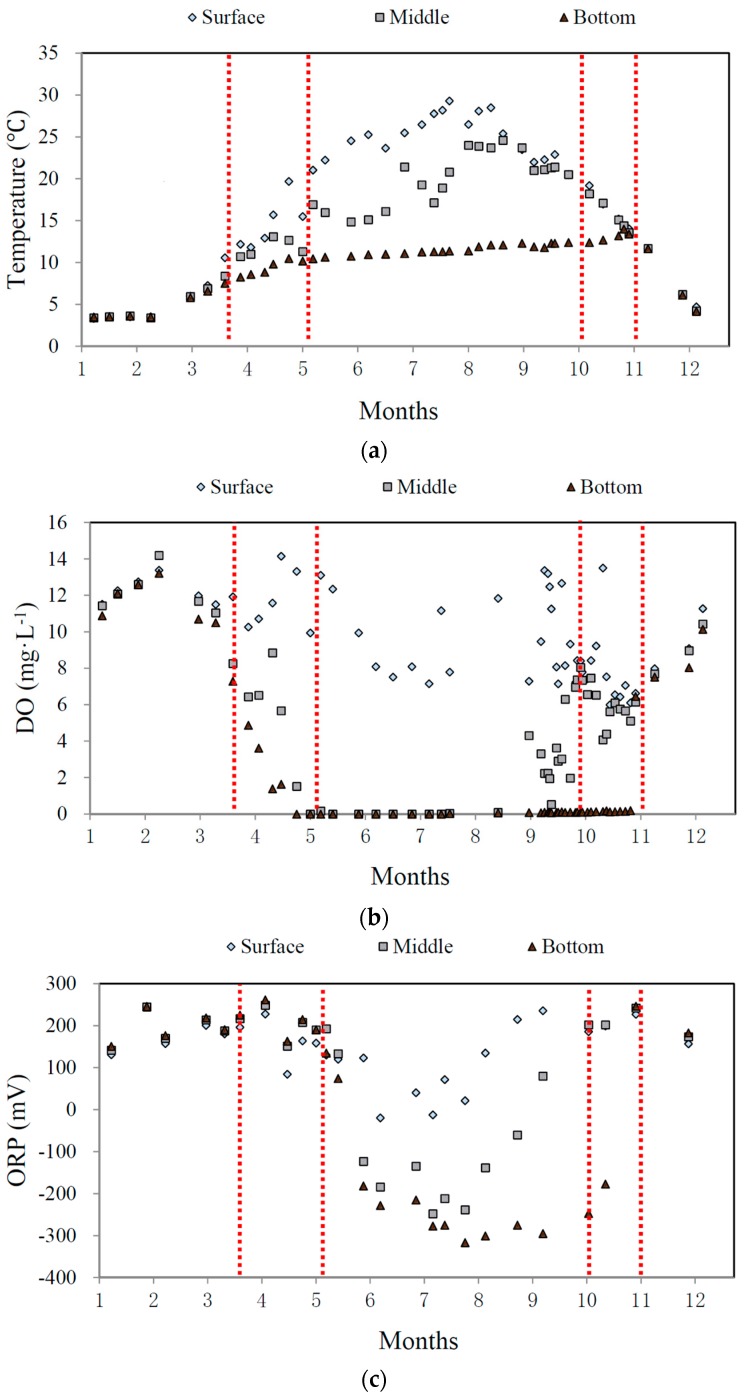
Variation of temperature (**a**); DO (**b**); and ORP (**c**) in different water layers in the Zhoucun Reservoir.

As shown in Figure 6a, in Zhoucun Reservoir there is obvious seasonal variation in water temperature gradient. By the temperature difference between the surface and lower layers, the temperature distribution in the reservoir can be divided into three stages. From November to mid-March of the following year, water temperature is uniform in the reservoirs (the mixing period); from mid-May to September is the stratified period, when the temperature difference between the surface and lower water is more than 10 °C. For the rest of the year, the temperature difference is between 0–10 °C, and this period can be regarded as the transitional period between the mixing and stratified periods. The water stability index RWCS (Figure S1, Supplementary Materials) was used to indicate the thermal stratification. The RWCS varies from 0–20, 20–200, and 200–500 in the mixing, transitional, and stratified periods, respectively. 

The DO values show “aerobic-anaerobic-aerobic” state changes in the bottom and middle water layers during the whole year. Affected by thermal stratification, DO in the three water layers exhibit a remarkable difference. The level of DO has a strong influence on the formation of sulfides. Reduction of sulfate in sediments without dissolved oxygen can be described by Equation (3) [36]:

(3) SO_4_^2−^ + 2CH_2_O + 2H^+^→H_2_S + 2CO_2_ + 2H_2_O

The seasonal variation of DO is shown in Figure 6b. During the stratified period starting in mid-May, DO is exhausted (DO = 0 mg·L^−1^) in the bottom and middle layers of water. However, ORP at the same time, shown in Figure 6c, indicates that the water in the whole reservoir is in the oxidized state. This is due to the presence of chemical substances with high oxidization potentials, such as MnO_4_^−^, NO_3_^−^, and Fe^3+^. Considering the half-reactions listed in Table S1 (Supplementary Materials), in the sediments the reduction of sulfate without dissolved oxygen is more difficult than the reduction of those high potential substances; therefore, sulfides remained undetectable until mid-June when the ORP was lower than 0 mV.

As shown in Table 1, the correlation of sulfides with RWCS (*r* = 0.70; *p* < 0.01) and ORP (*r* = −0.84; *p* < 0.01) is significantly higher than the correlation of sulfides with DO (*r* = −0.50; *p* < 0.05). This result led us to conclude that thermal stratification affects DO levels in water especially in the bottom layer, and the lower ORP results in the release of sulfides and VOSc from the sediments.

### 3.3. In Situ Release of Sulfides and VOSc in Sediments 

Sulfides pollution is mainly concentrated in the lower water. According to the research of Yang [37], the freshwater habitat of the Zhoucun Reservoir shows a relative abundance of SRB, which is similar to the observation in marine sediments. A SRB count of approximately 4.50 × 10^4^ cells/g sediment was estimated from the samples taken in 2013, which is a magnitude greater than in typical reservoirs. Analysis indicated that this is due to the accumulation of nutrients from cage fish aquaculture, which produced conditions in the sediments similar to those in marine sediments. Therefore, these pollutants were believed to originate from the sediments of the reservoir.

To reproduce the sulfide releasing process, an *in situ* experiment was carried out in 2014. Based on the experimental data, the calculated rate of oxygen consumption by the sediments in the Zhoucun Reservoir is 15.80–22.60 mg·m^−2^·h^−1^, with an average of SOD = 19.20 mg·m^−2^·h^−1^ in a different time. According to this result, when the water becomes thermally stratified in the summer, the rate of oxygen consumption by the sediments is high enough to reduce DO to zero, creating an anaerobic condition in the lower water layer.

**Figure 7:**
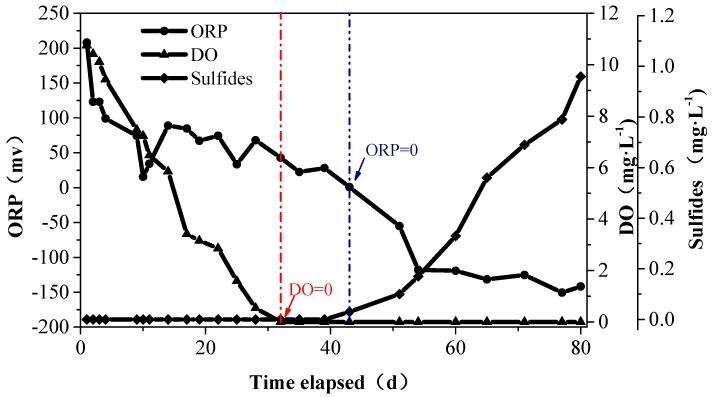
The variation of sulfides, DO, and ORP with the reaction time in the experimental chamber.

As shown in Figure 7, on the 32nd day of the experiment DO was exhausted, but the sulfides concentration in the chamber stayed at the same level as that at the beginning. When the ORP was down to 0 mV on the 43rd day, the sediments started to release sulfides. During the 10 days after the onset of DO exhaustion ORP decreased gradually, which is due to the reduction of highly-oxidizing species (e.g., MnO_4_^−^, NO_3_^−^, Fe^3+^). The reduction of SO_4_^2−^ led to a sharp decline of ORP, from 0 to −130 mV in 20 days as observed in Figure 7. During the experiment, the concentration of sulfides rose from 0 to 0.96 mg·L^−1^ in the chamber. 

These experimental results confirmed the conclusion in Section 3.2 about the sulfide release condition from the sediments (ORP = 0 mV). The disruption of thermal stratification and anaerobic environment is critical for controlling the release of sulfides and VOSc.

### 3.4. Control of Sulfides and VOSc Using Water-Lifting Aeration System

#### 3.4.1. The Distribution of DO around WLA

**Figure 8:**
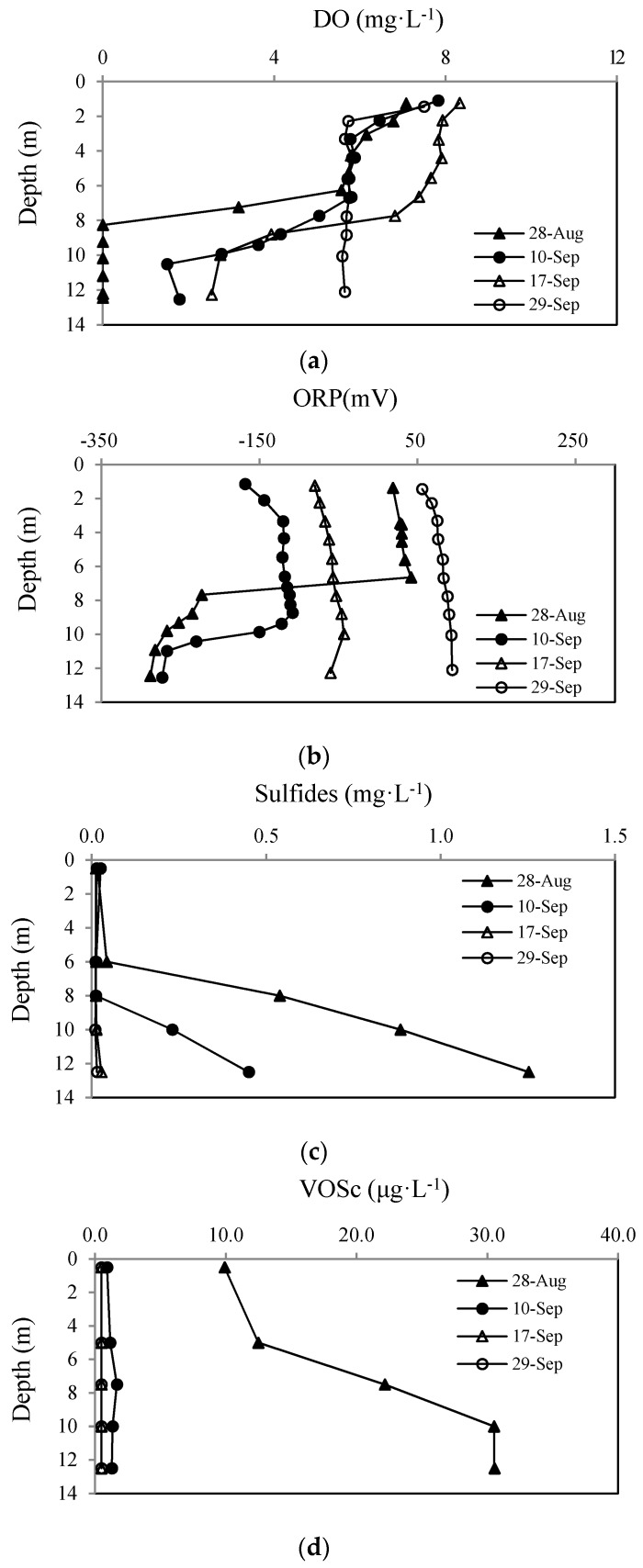
Vertical distributions of DO (**a**); ORP (**b**); sulfides (**c**); and VOSc (**d**) at 50 m from the Water-lifting Aerator.

The DO’s vertical distribution around the WLA is shown in Figure 8a. Before the operation of WLA, the DO concentration at the sediment surface was 0 mg·L^−1^ while its value at the water surface was high (>7.00 mg·L^−1^). This indicates that the sediments’ oxygen consumption caused the rapid decrease of DO in the lower water layer. The variation in DO concentration from the surface to 6 m above the sediment is insignificant (from 7.10 to 6.00 mg·L^−1^). Yet, the DO changed greatly within the lowest 6 m above sediments (from 6.00 mg·L^−1^ to 0 mg·L^−1^ at sediment surface). Therefore, without external mixing, the diffusion of dissolved oxygen from the surface to the lower layer is difficult.

As the WLAs started to operate, an obvious change occurred in the vertical distribution of DO. The bottom water DO concentration was up to 1.8 mg·L^−1^ after 14 days of WLA operation. DO in the upper layer rose to 7.40–8.30 mg·L^−1^, while it was 2.50 mg·L^−1^ in the bottom layer. After 32 days of operation, no difference of DO between upper and lower water layers was observed. The DO was 5.6 mg·L^−1^ in the bottom layer, while its values in the upper and middle layers were slightly decreased due to the mixing of water from the lower layer.

In summary, the difference in DO concentration between the upper and lower layers was eliminated through aerating and mixing by WLA. Clearly, the water-lifting aeration system can improve DO concentration in the lower layer in a relatively short time.

#### 3.4.2. Variation of ORP near the WLA

Figure 8b shows the variation of ORP during WLA operation. Before the operation of WLA, ORP in the water was −288 and 40 mV at the bottom and surface layers, respectively, with a thermocline at 6 m below water surface, which divided the reductive environment at the bottom from the oxidative environment at the top. On the 14th day of WLA operation, ORP in the surface water decreased to −110 mV due to the mixing of upper and lower water layers, which brought reductive substances in the bottom up to the surface. From this time on, ORP rose gradually, and all layers of water was in the oxidative condition after 32 days of operation. 

According to the discussions in Section 3.2 and Section 3.3, the release of sulfides from sediments is inhibited in the oxidative environment, which occurred about two months ahead of the natural changes (Figure 6c). With the coming of winter, the air temperature would reduce and the reservoir surface water temperature would continue to decline, thus, the density of the surface water would be increased. The low temperature, high density, and oxygen-rich surface water would continuously be transferred to the lower layer, maintaining a continuous oxidative environment at the lower layer water until June of the next year.

#### 3.4.3. Sulfides and VOSc Distribution near the WLA

The vertical distribution of sulfides 50 m away from WLA is shown in Figure 8c. Before WLA operation, the sulfides concentration in the lower layer was much higher than that in the upper layer. During WLA operation the sulfides concentration in the lower layer gradually decreased, with the highest rate of decrease at the beginning of the operation. The sulfides concentration in the upper layer rose slightly during the same time, and the concentrations in the upper and lower layers became closer to each other. After 21 days of operation, the sulfides concentration in the lower layer decreased from 1.25 to 0.02 mg·L^−1^, and changed slightly in the upper layer from 0.01 to 0.02 mg·L^−1^.

Figure 8d shows the variation of vertical distributions of VOSc concentration 50 m away from the WLA. Due to the volatile properties of VOSc, they are easily removed from the water by mixing. Therefore, the VOSc concentration decreased rapidly when the WLAs started to operate, becoming undetectable at the end of the oxygenation operation. These results show that sulfides and VOSc in the water were effectively removed by the WLA operation, with a reduction rate up to 98%.

#### 3.4.4. Principles of Water Quality Improvement Using Water-Lifting Aeration System

Water-lifting aeration systems directly oxygenate and mix water. As the bottom layer has low DO concentration and compressed air is released into the water by WLA at high pressure, the oxygen transfer efficiency is quite high. In thermally-stratified lakes and reservoirs, when water in the lower layer is transported to the higher one, the mixed water is still heavier than the surface layer water, so the mixed water will slowly sink. DO in the lower layer water can be increased by either direct oxygenation or transport of oxygen-rich water from the upper layer by vertical circulation. Once the anoxic condition in the lower layer is destroyed by increased DO, the dissolution and release of sulfides and other reductive substances from the sediments under anaerobic reductive conditions will be inhibited [38].

Under the stratified period, the diffusion of H_2_S and VOSc from water to the atmosphere is slow. Therefore, they can accumulate in the lower layer, and pose a threat to aquatic organisms. The mixing can destroy temperature stratification and, thus, promote the diffusion of the dissolved volatile substances. On the other hand, the air piston from the WLA can carry the dissolved H_2_S and VOSc to the surface and then strip them to the atmosphere. Therefore, sulfides and VOSc in the water as well as their associated foul smell are effectively eliminated. Furthermore, oxygenation of the water by WLA eventually led to the rise of ORP, and the resulting oxidative environment inhibited the release of sulfides from sediments.

The operation of WLA led the reservoir to be mixed in advance with an increased vertical mixing temperature. The low-temperature, high-density, and oxygen-rich surface water would be continuously transferred to the lower layer, forming a continuous mixing process in the natural state. Thus, good water quality could also be maintained after the WLA stopped running. The operation of WLA could effectively restrain the release of N/P/S from the sediments and algal growth was inhibited, thus reducing the cost of drinking water treatment and ensuring the safety of drinking water for urban residents.

Compared to other traditional methods of water quality improvement, such as enhanced water treatment processes in water utilities, which is unstable and passive, water-lifting technology can preemptively prevent the problems from arising, has no side effect, and is generally applicable in lakes and reservoirs.

## 4. Conclusions

The increase of sulfide concentration in Zhoucun Reservoir began in the middle of June, with the highest concentration in August and September (peak value 1.59 mg·L^−1^) 2014. The concentration of VOSc correlates with that of sulfides during the summer, with a peak VOSc concentration of 44.37 μg·L^−1^ during the same observation period. The main cause of elevated sulfide levels in the reservoir is endogenous pollution. Thermal stratification causes DO to decrease in water, especially in the bottom layer. When the ORP value is down to 0 mV, sulfides and VOSc are released from the sediments. The water-lifting aeration system can oxygenate the lower layer water directly, and mix water among the layers. The mixing can quickly and effectively remove dissolved sulfides and VOSc. Oxygenation of the water by WLA also leads to the rise of ORP level, thereby inhibiting the release of sulfides from sediments.

## Figures and Tables

**Table 1:** Pearson correlation (*r*-value) matrix for water quality parameters.

Parameter	DO	ORP	RWCS	Sulfides
DO	1			
ORP	0.66 **	1		
RWCS	−0.77 **	−0.80 **	1	
Sulfides	−0.50 *	−0.84 **	0.70 **	1

* *p* < 0.05; ** *p* < 0.01; positive values indicate positive correlation; negative values indicate negative correlation; *n* = 25.

## Supplementary Materials

The following are available online at www.mdpi.com/1660-4601/13/4/427/s1, Figure S1: Variation of RWCS in Zhoucun reservoirs, Table S1: Relevant half-reaction in the sediments, Table S2: Data for VOSc and sulfides measurements, Table S3: Regression equation of compound (VOSc) determined in the water by GC-MS.

## Abbreviations

The following abbreviations are used in this manuscript:

VOSc: Volatile organic sulfur compounds
WLA: Water-lifting aerator
DO: Dissolved oxygen
ORP: Oxidation reduction potential
SOD: Sediments oxygen demand
