# Supplementary material for: The Variation Characteristic of Sulfides and VOSc in a Source Water Reservoir and Its Control Using a Water-Lifting Aerator
Source: Int J Environ Res Public Health. 2016 Apr 15;13(4):427. doi: 10.3390/ijerph13040427 (PMC4847089; doi:10.3390/ijerph13040427)
Supplement: Supplementary file 1 [file ijerph-13-00427-s001.pdf]

Supplementary Materials: The Variation Characteristic of Sulfides and VOSc in a Source Water Reservoir and Its Control Using a Water-Lifting Aerator

Jian-Chao Shi, Ting-Lin Huang, Gang Wen, Fei Liu, Xiao-Peng Qiu and Bao-Shan Wang

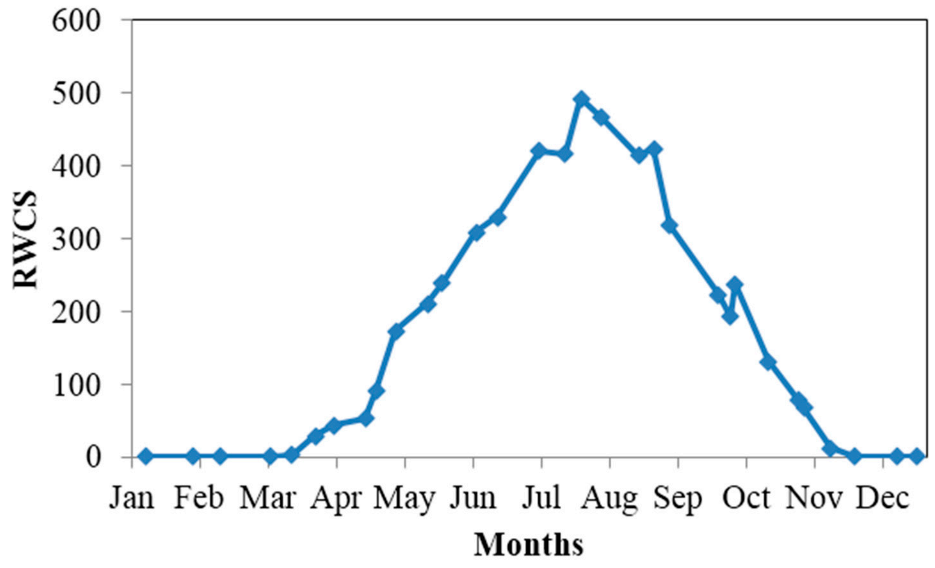

Figure S1. Variation of Water Stability Index (RWCS) in the Zhoucun Reservoirs.

TableS1. Relevant half-reaction in the sediments.

Half-Reaction Equation	Eh (V)
$0.5O_2 + 2H^+ + 2e^- = H_2O$	+0.82
$MnO_4^- + H^+ + e^- = HMnO_4^-$	+0.90
$NO_3^- + 2H^+ + e^- = NO_2^- + H_2O$	+0.80
$Fe^{3+} + e^- = Fe^{2+}$	+0.77
$SO_4^{2-} + 10H^+ + 8e^- = H_2S + 4H_2O$	-0.22

Table S2. Data for VOSc and sulfide measurements.

Date	Surface (0.5 m)		Middle (7.5 m)		Bottom (15 m)	
	Sulfides *	VOSc **	Sulfides	VOSc	Sulfides	VOSc
10 January	0.00	0	0.01	0.70	0.01	1.27
15 February	0.00	0	0.00	0	0.00	1.23
2 March	0.00	0	0.00	0	0.01	1.58
1 April	0.00	0	0.01	0	0.02	1.34
3 May	0.01	0.60	0.01	0.60	0.02	1.29
1 June	0.01	1.50	0.04	2.61	0.12	7.86
1 July	0.02	6.36	0.11	16.26	0.49	21.74
19 August	0.02	18.59	0.51	24.84	0.92	44.37
2 September	0.01	11.19	0.61	24.31	1.59	32.89
6 October	0.01	5.22	0.41	19.28	0.87	24.27
11 November	0.02	2.16	0.01	5.29	0.01	13.01
13 December	0.00	0.74	0.01	1.83	0.02	6.21

* Sulfides in $mg \cdot L^{-1}$; ** VOSc in $\mu g \cdot L^{-1}$.

Table S3. Regression equation of compound (VOSc) determined in the water by GC-MS.

Compound	Retention Time (min)	Characteristic Ion (*m*/*z*)	Regression Equation	R^2
Dimethyl sulfide	3.1	62.47	y = 11,108 x − 10,629	0.9988
Dimethyl disulfide	5.3	94.79	y = 15,270 x + 55,610	0.9965
Ethanethiol	6.7	108.80	y = 14,770 x + 46,720	0.9994
Diethyl disulfide	7.8	122.66	y = 16,500 x + 30,470	0.9972
Dimethyl trisulfide	8.5	126.79	y = 1775 x − 18,010	0.9951

© 2016 by the authors; licensee MDPI, Basel, Switzerland. This article is an open access article distributed under the terms and conditions of the Creative Commons by Attribution (CC-BY) license (http://creativecommons.org/licenses/by/4.0/).
